# Characterization and Prediction of Cracks in Coated Materials: Direction and Length of Crack Propagation in Bimaterials

**DOI:** 10.1155/2015/594147

**Published:** 2015-01-31

**Authors:** C. I. Pruncu, Z. Azari, C. Casavola, C. Pappalettere

**Affiliations:** ^1^Mechanical Engineering, University of Birmingham, Edgbaston, Birmingham B15 2TT, UK; ^2^LaBPS, Université de Lorraine, Ecole Nationale d'Ingénieurs de Metz (ENIM), 1 Route d'Ars Laquenexy, 57070 Metz, France; ^3^Dipartimento di Meccanica, Matematica e Management, Politecnico di Bari, Viale Japigia 182, 70126 Bari, Italy

## Abstract

The behaviour of materials is governed by the surrounding environment. The contact area between the material and the surrounding environment is the likely spot where different forms of degradation, particularly rust, may be generated. A rust prevention treatment, like bluing, inhibitors, humidity control, coatings, and galvanization, will be necessary. The galvanization process aims to protect the surface of the material by depositing a layer of metallic zinc by either hot-dip galvanizing or electroplating. In the hot-dip galvanizing process, a metallic bond between steel and metallic zinc is obtained by immersing the steel in a zinc bath at a temperature of around 460°C. Although the hot-dip galvanizing procedure is recognized to be one of the most effective techniques to combat corrosion, cracks can arise in the intermetallic *δ* layer. These cracks can affect the life of the coated material and decrease the lifetime service of the entire structure. In the present paper the mechanical response of hot-dip galvanized steel submitted to mechanical loading condition is investigated. Experimental tests were performed and corroborative numerical and analytical methods were then applied in order to describe both the mechanical behaviour and the processes of crack/cracks propagation in a bimaterial as zinc-coated material.

## 1. Introduction

The knowledge of mechanical materials behaviour is of great importance particularly for some innovative engineering applications, which involve a single material, bimaterials, or multiple materials. Some bimaterials are obtained by means of a coating process and consist of coupled layers that may or may not contain filler elements, such as a foaming operation, or may be developed as welded substrates. Usually, at the end of the process, the material is identified as a new single material. The coating procedure developed and studied in this work refers to the hot-dip galvanizing treatment, considered as a better treatment to fight against corrosion.

**Figure 1:**
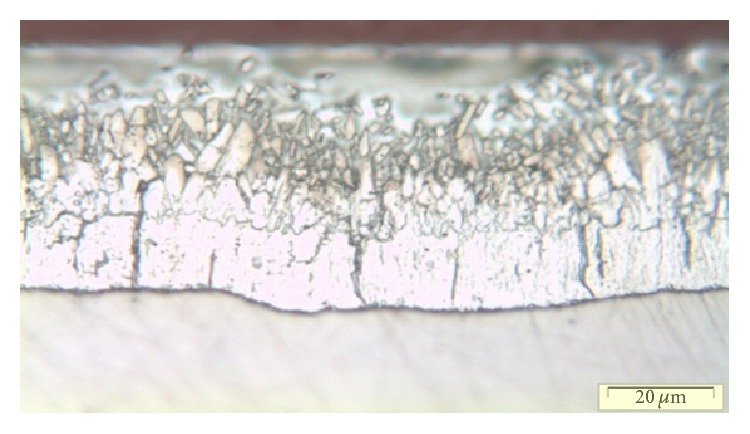
Coated product as bimaterials after bath at 460°C.

The role of a hot-dip galvanizing treatment consists in the deposition of a protective external layer of metallic zinc obtained by immersing the steel in a zinc bath at a temperature of around 460°C. When the material (i.e., steel) is introduced into the zinc bath and then removed, several changes in the chemical composition and in the mechanical structure can occur. These changes produce a new structural arrangement on zinc substrate and are usually revealed by the generation of cracks in the zinc layer. The presence of such cracks is illustrated in Figure 1.

In practice, the behaviour of materials may change as a result of various events such as fatigue, fracture, wear, fretting fatigue, creep, hydrogen embrittlement, thermal shock processes, or atmospheric attacks. When corrosion is involved, the life of materials decreases considerably. An important role in these failure processes is played by any discontinuity in the material that can appear during the manufacturing process or during working period, or as a result of inappropriate use. In our case it appears as a consequence of the hot-dip galvanizing treatment applied as described above in association with the action of the mechanical loading.

Powerful concepts have been advanced in literature to clarify the behaviour of bimaterials. Recently, Krishnan and Xu [1] focused on failure mechanics of adhesive joints (i.e., considered as bimaterials) using a fringe pattern concentrations technique to describe the bimaterials interface. As analytical model, they used the fracture mechanics approach, while to compute the stress singularity in the bimaterial layer they took into account the stress intensity factor in Mode I. The formula used to express this stress intensity factor is

(1) KI=ReKaiε,

where *K* is the stress intensity factor in the case of the bimaterial component:

(2) $$\begin{array}{c} K=YT\sqrt{a}a^{-i\varepsilon }e^{i\psi }, \end{array}$$

where *T* = *P*(3*S*/*W* ^2^) and *Y*, *ψ* are the calibrating factors that depend on *a*/*W*, *B*/*W*, respectively, Dundurs' parameters. Then *a*, *B*, and *W* represent the length of the possible crack, the thickness, and the length of the specimen. *ε* is a function of Dundurs' parameters, *β*, and is presented as

(3) $$\begin{array}{c} \varepsilon =\frac{1}{2\pi }\ln\left\{\frac{1-\beta }{1+\beta }\right\}, \end{array}$$

where *β*, Dundurs' parameters material, is expressed by

(4) $$\begin{array}{c} \beta =\frac{\mu_{1}\left(1-2\upsilon_{2}\right)-\mu_{2}\left(1-2\upsilon_{1}\right)}{\mu_{1}\left(1-2\upsilon_{2}\right)+\mu_{2}\left(1-2\upsilon_{1}\right)} \end{array}$$

in which 1 and 2 denote the material, while *υ* and *μ* are the shear modulus and Poisson's ratio, respectively.

The elastic fracture theory is a suitable tool to compute the mechanical behaviour in case of coatings, composites, and welded structures. The bimaterial produced by the reactions that occur during the hot-dip galvanized steel process must have the same conditions on the contact surface as the “linear spring-like” model. The discontinuity of displacement across the interface is assumed to be linearly proportional to the displacement at the interface of the constituent where the stress source is located.

The present authors agree that the two materials that form the bimaterial should be considered as a functionally autonomous subsystem with a different Young's modulus and Poisson's coefficients. This implies a strain incompatibility between the two solids and the formation of a periodic distribution of tensile and compressive stress in checkerboard patterns under the uniaxial tensile test.

A robust approach was proposed by Yu et al. [2] in order to formulate analytical solutions through the use of “linear spring-like model.” According to this theory we can define the type of contact between the surfaces by the fraction of the adherent area, applying the following formula:

(5) $$\begin{array}{c} \gamma =\frac{A-A_{c}}{A_{c}}, \end{array}$$

where *γ* is used to quantify the extent of bonding at the interface, *A* is the total area of the interface, and *A*_*c*_ is the area covered by paper (see example on Figure 2 from [2]).

According to the study made by Panin et al. [3], the interface of solid materials covers a sinusoidal surface layer due to a rotation of successive constraints in tensile and compression stress in the structure. This effect is described by the following equation:

(6) $$\begin{array}{c} \sigma =A\sigma_{y}\sin\frac{x-l_{x}}{t\sqrt{2}}, \end{array}$$

where “*t*” is the thickness of the coating, *x* is the distance of crack propagation, *σ*_*y*_ is the stress coefficient.

## 2. Objectives

This paper addresses two goals: first of all, to experimentally characterize the layer of zinc applied during the hot-dip galvanization process, in order to obtain necessary information about the uniformity of the zinc layer and the number of cracks and their length, and finally to estimate the behaviour of the cracks located in zinc layer when the mechanical loading is imposed. Secondly, we proposed to corroborate the experimental results with analytical and numerical computations, ascertaining where the crack discontinuities spread while considering the three main possibilities of crack development: arrest at the bimaterial interface, crossing into the second material, in our case steel, and finally creating a deflection between the two materials.

**Figure 2:**
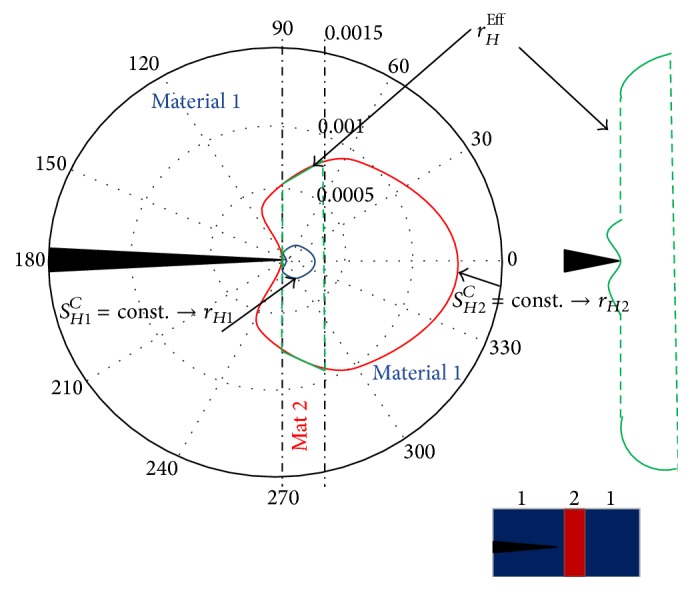
Diagram of the damage areas, according to the decohesion criterion for an SM-WI boundary specimen (Figure taken from [4]).

In the literature, different approaches have been adopted to address this issue. K. M. Mróz and Z. Mróz [4] predicted the behaviour of bi- and multimaterial interfaces according to a simplified approach using the MK-criterion based on the linear elastic fracture mechanics (LEFM). In this criterion it is assumed that the crack growth follows the direction of minimum distortion energy density at a distance corresponding to a specified value of dilatation energy.  Figure 2 shows a specific case in which the crack is considered to propagate perpendicularly to the interlayer and make a bifurcation at the interface.

The decohesion phase may present as one of the four possible scenarios whereby the crack will propagate as follows: (i) the plastically weaker material (WM) to the plastically stronger material (SM), WM-SM, (ii) the plastically weaker material (WM) to the plastically stronger interlayer (SI), WM-SI, (iii) the plastically stronger material (SM) to the plastically weaker material (WM), SM-WM, and (iv) the plastically stronger material (SM) to the plastically weaker interlayer (WI), SM-WI.

In simple terms, the criterion for crack initiation and propagation along the interface could be considered, where the maximum stress, *σ*_max⁡_, is expressed as

(7) $$\begin{array}{c} \sigma_{\max}=\frac{\sigma_{x}+\sigma_{y}}{2}+\sqrt{{\left(\frac{\sigma_{x}+\sigma_{y}}{2}\right)}^{2}+\tau_{xy}.} \end{array}$$

To solve the problem of deflection at the interface of two materials, Hutchinson [5] proposed the following condition:

(8) $$\begin{array}{c} \frac{G_{c}\left(\psi \right)}{{G_{c}}^{\left(1\right)}}<\frac{G_{d}\left(\psi \right)}{{G_{p}}^{\left(1\right)}}, \end{array}$$

where *G* = *G*_*C*_ represents release energy rate of the interface bimaterial.

Another theory described in Madani et al. [6] studies, related to the problem of cracks running perpendicular to a bimaterial, yields a different expression for the stress intensity factor that is connected to the stress tensor as follows:

(9) $$\begin{array}{c} \sigma_{ij}=\frac{K}{r^{1-\lambda }}f_{ij}\left(\theta \right). \end{array}$$

To determine the propagation energy of the crack running perpendicular to the interface, also called energy release rate, the following equation studied by Madani et al. [6] can be applied:

(10) $$\begin{array}{c} G_{d}=\frac{\left[\left(1-\upsilon_{1}\right)/\mu_{1}+\left(1-\upsilon_{1}\right)/\mu_{2}\right]}{4\cosh^{2}\pi \varepsilon }\left(K_{1}^{2}+K_{2}^{2}\right)\\ G_{p}=\frac{1-\upsilon_{2}}{2\mu_{2}}K_{p}^{2}. \end{array}$$

In (8), (10)  *G*_*d*_ and *G*_*p*_ are the strain energy release rate for deflection and penetration; *K*_1_ and *K*_2_ are the stress intensity factors for the interface crack; *K*_*p*_ is the value of stress intensity factor, for a particular case, when the crack from material 1 penetrates into material 2 (see Figure 2).

Analytical techniques and the Green function can be employed to describe the behaviour of bimaterial compounds by calculating numerical solutions of singular integral equations, as was applied by Erdogan et al. [7] and used by Pruncu et al. [8] and Azari et al. [9], which yield the following expression:

(11) $$\begin{array}{c} K_{I}\left(A\right)=\frac{2\cdot \mu_{1}}{\left(1+k_{1}\right)}\sqrt{a_{0}}g\left(-1\right)\\ K_{I}\left(B\right)=-\frac{2\cdot \mu_{1}}{\left(1+k_{1}\right)}\sqrt{a_{0}}g\left(1\right). \end{array}$$

A more general form of such approach could be structured using an algorithm that describes the numerical procedure for obtaining the different values of the function *g*(*t*_*i*_) by the following.

(1) We divide the crack in *n* parts.

(2) For *k* ranging from 1 to *n*, we need a computing route for *x*_*k*_ and *f*(*x*_*k*_).

(3) For every *x*_*k*_ and for *i* variants from 1 to (*n* − 1), we have to calculate the first *t*_*i*_ that is a weight function of the Jacobi polynomials described as follows:

(12) 1n×g(ti)×1ti−xk+π×k(xk,ti)=0 withti=cos⁡⁡π·2·i−12·n, i=1,…,nxk=cos⁡⁡π·kn, k=1,…,n−1.

(4) The computation then yields a matrix relationship expressed as follows:

(13) $$\begin{array}{c} \left[\begin{array}{ccccc} \left[\frac{1}{t_{1}-x_{1}}+\pi \cdot k\left(x_{1},t_{1}\right)\right] & \left[\frac{1}{t_{2}-x_{1}}+\pi \cdot k\left(x_{1},t_{2}\right)\right] & .. & .. & \left[\frac{1}{t_{n}-x_{1}}+\pi \cdot k\left(x_{1},t_{n}\right)\right]\\ .. & .. & .. & .. & ..\\ .. & .. & .. & .. & ..\\ .. & .. & .. & .. & ..\\ \left[\frac{1}{t_{1}-x_{k}}+\pi \cdot k\left(x_{k},t_{1}\right)\right] & \left[\frac{1}{t_{2}-x_{k}}+\pi \cdot k\left(x_{k},t_{2}\right)\right] & .. & .. & \left[\frac{1}{t_{n}-x_{k}}+\pi \cdot k\left(x_{k},t_{n}\right)\right] \end{array}\right]\cdot \left\{\begin{array}{c} g\left(t_{1}\right)\\ g\left(t_{2}\right)\\ ..\\ ..\\ g\left(t_{n}\right) \end{array}\right\}=\left\{\begin{array}{c} f\left(x_{1}\right)\\ f\left(x_{2}\right)\\ ..\\ ..\\ f\left(x_{k}\right) \end{array}\right\}. \end{array}$$

(5) Then the values of the vector “*g*” are obtained by multiplying the transposed of the matrix by the vector *f*(*x*_*i*_).

One has the following: *a*_0_ is half-length of the crack; *μ*_1_, *μ*_2_ are shear modulus for materials 1 and 2; *c* is the distance from the middle of the crack at the interface plane; *r*, *θ* are polar coordinates, *k* = 3 − 4*ν* for the plane strain case and *k* = (3–*ν*)/(1 + *ν*) for the plane stress case, *ν* is Poisson coefficient, and *g* is the parameter that expresses the magnitude of the applied load.

Another analytical technique, which was employed by Chen et al. [10] using complex potential values, was obtained in the following manner:

(14) $$\begin{array}{c} K_{I}\left(B\right)=\underset{r\to 0}{\lim}\sqrt{2\pi r}\sigma_{x}=-\frac{2\cdot \mu_{2}}{k_{2}+1}\sqrt{\pi a_{0}}\overset{\infty }{\underset{m=1}{\sum }}{\left(-1\right)}^{m}\alpha_{m}\\ K_{I}\left(A\right)=-\frac{2\cdot \mu_{2}}{k_{2}+1}\sqrt{\pi a_{0}}\overset{\infty }{\underset{m=1}{\sum }}\alpha_{m}, \end{array}$$

where *a*_0_ is half-length of the crack, *α*_*m*_ = *mπ*/*b*, *m* = 0,1, 2,3,…,  *b* is the distance between the interface and the crack front, *σ*_*x*_ is the distribution of stress in front of the crack, *k* = 3 − 4*ν* for the plane strain case and *k* = (3 − 4*ν*)(1 + *ν*) for the plane stress case, and *μ*_2_ is shear modulus for the material in which the crack is located.

## 3. Material and Experimental Procedures

Because of its multiple properties, steel is one of the most versatile materials used in industrial engineering applications. The European Standard (EN) reveals different types of steel employed in fields such as the automotive industry and aeronautical design. In this work we considered two steel alloys: HE360DR and S420MC. Their mechanical characteristics are summarized in Table 1. During the lifetime of service, in contact with the environment this metal may develop problems that could be prevented by applying the above-described process of hot-dip galvanization.

The specimens obtained after hot-dip galvanization were submitted in laboratory to fatigue tensile test in order to detect their behaviour under fatigue conditions conforming to real life and to observe the changes that occurred in the metallic zinc layer. The fatigue tests were performed on a servohydraulic testing machine capable of applying axial loads up to 100 kN, using a sinusoidal load wave, with a frequency of 30 Hz and a strength ratio *R* = 0.1.

After the fatigue test the specimens were experimentally analysed by optical microscopy and scanning electron microscopy (SEM). During observation with the optical microscope, a sample of length 5328 *μ*m was considered. To simplify the management of these optical microscope observations, we divided the sample size into 16 units with a length of about 333 *μ*m. The main purpose of this was to analyse (i) the number of cracks per unit of length; (ii) whether the evolution of the number of cracks was constant over all layers applied; (iii) which material was prone to develop longer cracks, and finally if the zinc layer was constant over the whole steel surface. The first result was the average number of cracks per unit of length, as summarized in Table 2.

**Figure 3:**
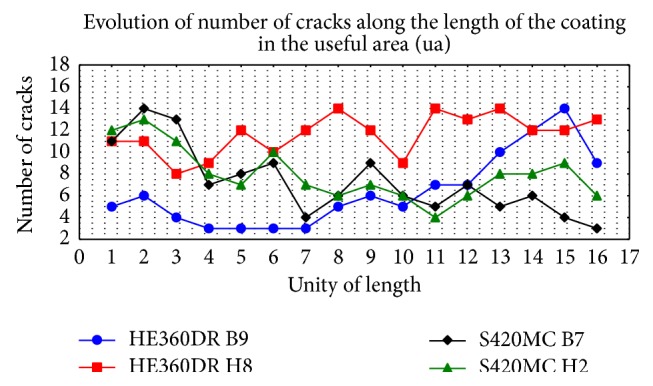
Number of cracks per unit of length in the full length sample (5328 *μ*m).

The letters B and H in Table 2 indicate the time of immersion in the zinc bath, B being 3 minutes of immersion and H 7 minutes. The numbers 9, 8, 7, and 2 indicate the specimen number analysed. From Table 2 we can observe that the average number of cracks per unit of length is about 6/11 for HE360DR and 7/8 for S420MC. A first remark proves that the number of cracks increases with the time of immersion. Anyway, it seems that a single unit did not provide enough information. So the observation was extended on full length specimens consisting of 16 units. The results are plotted in Figure 3 and highlight the number of cracks for all 16 units of length. Figure 3 shows that the number of cracks on the entire length of zinc layer that encloses each unit is overall scattered; however, on the local case of two/three consecutive units, the number of cracks per unit seems to be almost uniformly distributed.

**Figure 4:**
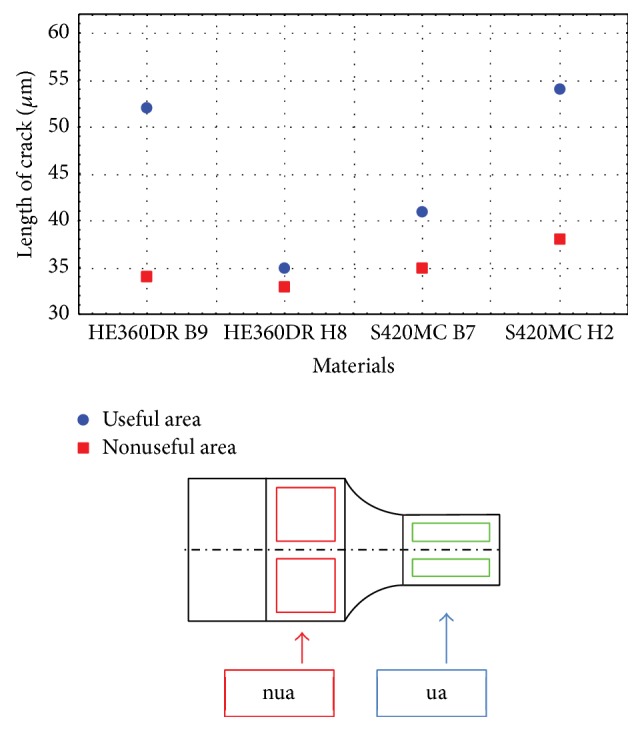
Length of cracks before and after the test in two distinct areas.

A key element for determination of the efficiency of the coating process may be deduced from the evolution of cracks lengths before and after the fatigue tests. According to the observations shown in Figure 4, there was a significant change of crack length as a consequence of fatigue test. A gap of about 25 *μ*m in crack length before and after cyclic load was found due to the weakened structure of material.

This observation was done in a useful sample area (ua) and then in the area where the test had less influence on the behaviour of the piece, marked as the nonuseful area (nua).

**Figure 5:**
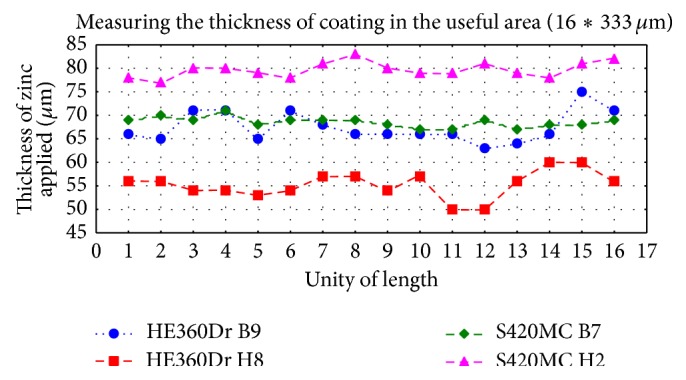
Evolution of the thickness on each unit of sample length.

The uniformity of the zinc layer was considered as another issue that can stimulate the crack behaviour. The results obtained are shown in Figure 5. In conformity with [8], the zinc layer deposited over the steel alloy is uniformly distributed, having a value of 50–80 *μ*m. The particularity of the size of substrate of HE360DR H8 materials, that is, the thin thickness, may be explained as a consequence of immersion time. So, because the time of immersion within the bath of zinc was less than the time imposed by our methodology, the thickness of substrate was lower; this means that instead of 7 minutes we found that the real time of immersion was about 5 minutes.

**Figure 6:**
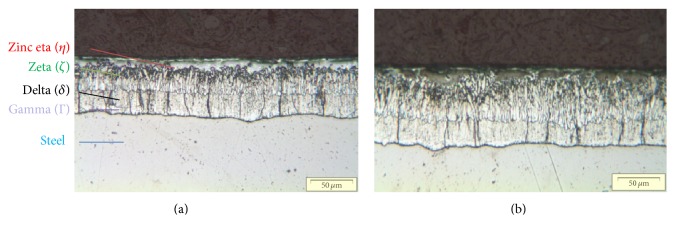
Layer substrate at optical microscopy for S420MC (3 minutes of immersion in zinc bath) and HE360DR (7 minutes).

The optical measurements confirmed that the thickness deposited over the steel pieces was uniform and show that the zinc layer is composed of several multilayers. The substrates are denominated by Gamma (Γ), Delta (*δ*), Zeta (*ζ*), and Zinc eta (*η*), as shown in Figure 6. Figure 6 proves that most of the cracks are located in substrate *δ* and may occur along the zinc grain boundaries [11]. Indeed, from our observations these cracks arise in this *δ* substrate and then spread toward the steel-zinc interface. However, the analysis indicates a decrease of the crack magnitude near the interface, which means the crack growth is interrupted before it reaches the interface.

**Figure 7:**
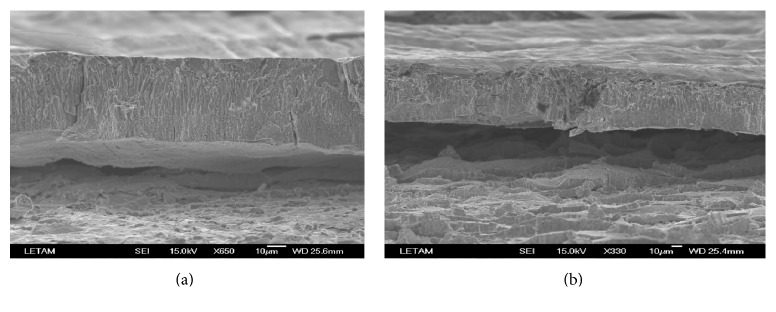
Debonding HE360DR (7 minutes of immersion in zinc bath) and S420MC (3 minutes).

Observations from scanning electron microscopy (SEM) exhibit even more clearly that, during the hot-dip galvanized process (HDG), the quality of the bonded layers may influence the direction of the cracks, if the cracks touch the steel-zinc interface. This troublesome problem produces a sort of “debonding area” which forms a path for surface deflection of the cracks. The “debonding area” in steel-zinc adhesive layers is illustrated in Figure 7, created by the propagation of cracks at the interface between the zinc and steel and into the zinc substrates zeta (*ξ*) and eta (*η*) [12]. For bimaterials, the interface crack problems could be due to the nonhomogeneity of the materials, which develop in the direction parallel to the crack tip.

From these preliminary findings we could confirm that during the HDG process the zinc layer is uniformly deposited and the average number of cracks is significant on this layer. It seems that during the fatigue process the cracks get longer and spread toward the bonding interface, arresting at the bimaterial interface. In the worst scenario, the cracks may make a bifurcation between the steel and zinc.

## 4. Simulation Procedure

Numerical computational technique was implemented by ABAQUS software, in order to corroborate the experimental data with analytical and numerical simulations and to highlight the effects of cracks that develop during the hot-dip galvanizing process.

In order to implement the experimental data in the numerical model, an average thickness of zinc layer of 80 *μ*m was considered. Besides, this value corresponds with the thickness measured under the S420MC H2 materials (see Figure 5), and it allows making the assumption that the crack/cracks imposed in our models will spread only toward the interface steel/zinc. If we consider a smaller thickness, for example, the size of layer measured under HE360DR B9 material, and we adopt in the numerical model the critical size of crack detected after the loading test (see Figure 4), it will be obvious that the crack will cross all the coating substrate. But this issue is far from our experimental observation, since we had observed that the crack growth is only toward interface steel/zinc. Experimental data were introduced in our numerical model as the thickness size of zinc layer (Figure 5), while the contour of zinc layer on the entire material surface is considered homogenous and uniform (Figure 5). An initial length of crack and the maximum length of crack (see Figure 4) were imposed as constraint in the numerical program. The principles of linear fracture mechanics (LFM) were implemented in the numerical model in order to explain behaviour of crack at the interface steel/zinc that is assuming that all the materials contain a minimum flow that will grow during the mechanical loading. So, considering the crack located in zinc substrate (Figure 5) that will develop perpendicular to the applied load, it is possible to denote this state by Mode I fracture. It should be noted that in the numerical model a mechanical loading of 500 MPa corresponding to an average of ultimate tensile strength of galvanized materials (see Table 1) was imposed. A value of about 30 *μ*m was selected as the initial length of the crack/cracks before propagation, in agreement with experimental data (Figure 4). However, we know that the crack is bounded by two fronts, *A* and *B*. In this model, we assumed that the front of crack *B* was static and so it could not propagate, whereas the only front of crack *A* would spread.

## 5. Numerical Results and Discussion

**Figure 8:**
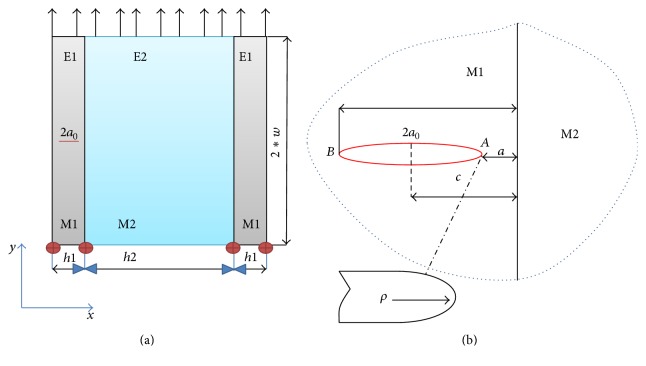
(a) Scheme of zinc-steel bimaterial body with a crack in the zinc layer (M1); (b) a finite crack perpendicular to the bimaterial interface of a finite solid.

A basic model composed of two materials, M1 and M2, as shown in Figure 8(a), which forms the bimaterial body, was implemented. The model proves the same characteristics as joined materials during the HDG process. M2 represents the steel and M1 the zinc layer, delimited by the following values: *h*1 = 0.08 mm, *h*2 = 19.84 mm, *c* = 0.045 mm, *w* = 15 mm, 2*a*_0_ = 0.030 mm, and *ρ* = 0.5 *μ*m and submitted to a mechanical load, *σ* = 500 MPa. The crack is present in material 1 (M1). The applied load in this system will have direct consequence to the crack behaviour and define the presence of stress singularity on both fronts of the crack, tips *A* and *B*. The configuration for the crack fronts is shown in Figure 8(b). Settlement of the crack propagates performance was evaluated using the stress intensity factor (SIF) parameter, derived from the singularity 1/*r* advanced in front of the crack tip. Since the value of the stress intensity factor (SIF) is obtained, we proceed to conclude if the crack crosses into the second material, ends at the bonded interface, or deflects between these two bodies.

Numerical simulation is implemented for two models.

**Figure 9:**
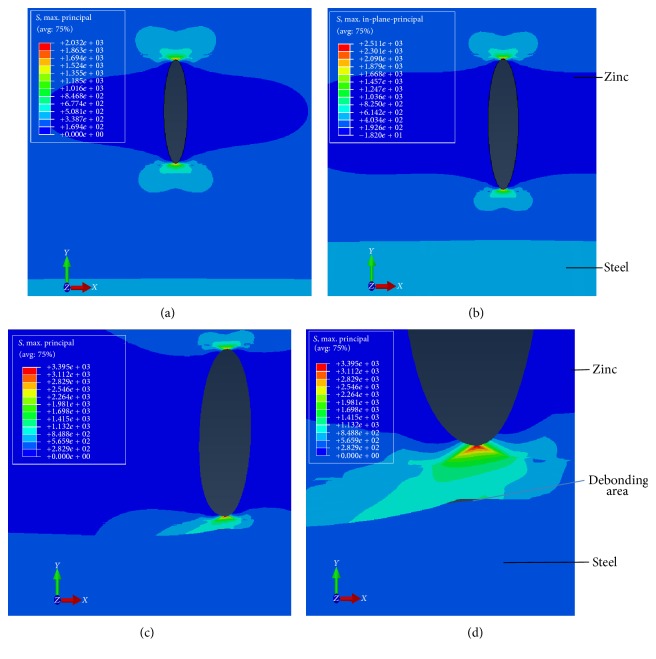
Results of FEM analysis with one crack and load of 500 MPa: (a) for a crack 30 *μ*m long; (b) the crack after propagation, now 57 *μ*m long; (c) and (d) when the crack forms a deflection between the steel and zinc.

(a) Model with one crack in the bimaterial: in this case one single crack of an initial size of 30 *μ*m was considered. Then the length was increased by 3 *μ*m in each simulation up to the maximum of 60 *μ*m (i.e., close to the experimental analysis shown in Figure 4). The maximum value was assigned when the crack reached the steel-zinc interface. The results are shown in Figure 9.

**Figure 10:**
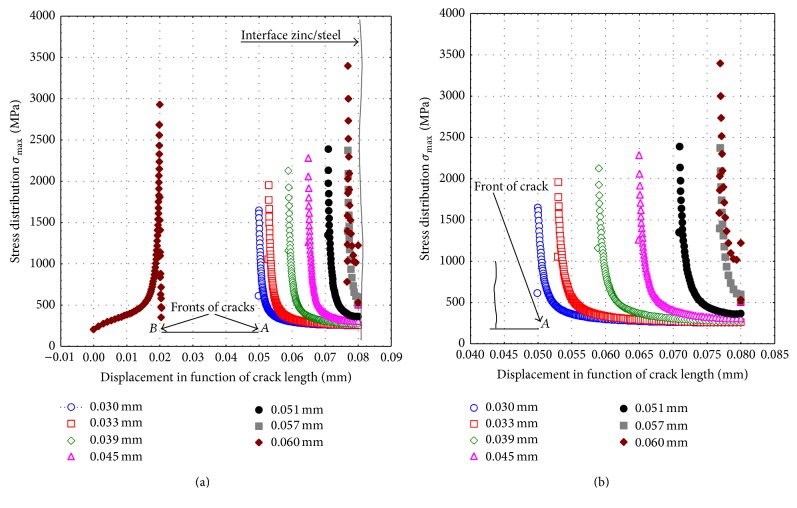
Stress-strain curve of the crack tip in front of cracks *A* and *B*, featuring different lengths.

Figure 9 shows how the stress distribution increases with the length of crack, assuming that the initial crack had the same stress value at both fronts *A* and *B*. Two-dimensional finite-element meshes are virtually symmetrical near this bottom (crack fronts) and the symmetry showed almost the same value as the stress distribution, for each of the crack fronts, during the simulation. The shape of the stress-strain curve as a function of the crack length is illustrated in Figure 10. The shape of the curve grows wide with the increasing of crack length in front of crack tip *A*.

The effect of stress distribution in front of crack can be converted into basic parameters that show the impact of crack behaviour, that is, the stress concentration factor (SCF). Obviously, the stress concentration factor is the ratio of the maximum stress over the nominal stress, denominated *k*_*t*_ and expressed as

(15) $$\begin{array}{c} k_{t}=\frac{\sigma_{\max}}{\sigma_{\mathrm{nom}}}. \end{array}$$

**Figure 11:**
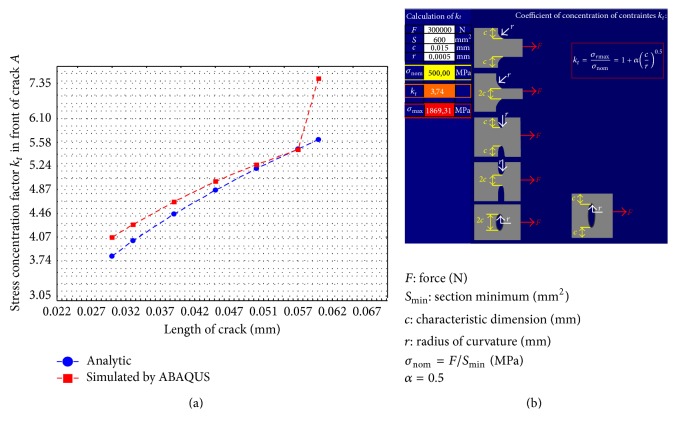
Distribution of the theoretical stress concentration factor versus length in front of crack tip *A*; (b) academic scheme for calculating the stress concentration factor *k*_*t*_ [13].

While the front of crack *B* does change, the main role in this research was played only by the front of crack *A* located near the interface of the bimaterial. Thus, Figure 11 shows the effect of SCF in the front of crack *A*. These results were corroborated with the theoretical stress concentration factor (SCF) obtained with the online software [13].

The results presented in Figure 11 show a good agreement and underline that when the crack reaches the interface, there is a sudden increase in the stress distribution value. This sharp rise in values may result in the creation of a surface called a “debonding area,” as shown in Figures 9(c) and 9(d).

**Figure 12:**
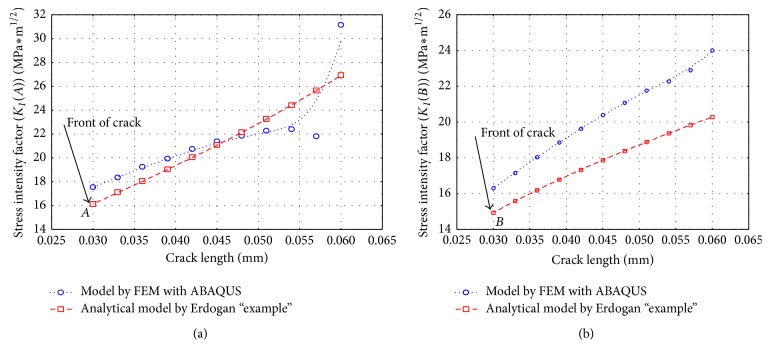
Value of the SIF in the bimaterial: (a) value of the crack tip in front of crack *A* during propagation and (b) value of the SIF in front of crack *B*.

Then, by assessing the crack propagation with LFM using (crack) stress intensity factors (SIF), *K*, it is possible to establish where the crack stops. To achieve even better accurate results, a comparison between an analytical model reported by Erdogan et al. [7] and the numerical results obtained by ABAQUS was accomplished. The distribution of the curve of the two models analysed shows a good correspondence. The difference between the numerical and analytical results is due to the use of a small number of constants in the analytical model. The results are shown in Figure 12, demonstrating the trend of the (crack) stress intensity factors (SIF) during applied stress, in front of cracks *A* and *B*. From the curves drawn below, it can be seen that the value of the SIF increases with the crack length, and the crack propagates up to a length of about 50 *μ*m. Then stabilization and even a decline of the SIF value can be observed. The value of SIF will increase sharply only if the flow area denoted as well as “debonding area” develops a high size. Since the value of the (crack) stress intensity factors (SIF) is low for the front of both cracks *A* and *B*, it seems that the crack cannot cross into the second material.

**Figure 13:**
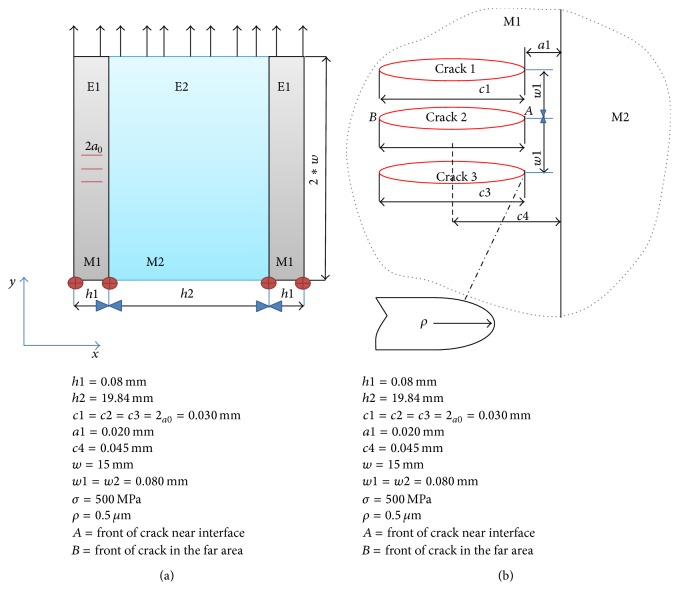
(a) Results for zinc/steel bimaterial body with 3 cracks in the zinc layer (M1); (b) three finite cracks perpendicular to the bimaterial interface of a finite solid.

**Figure 14:**
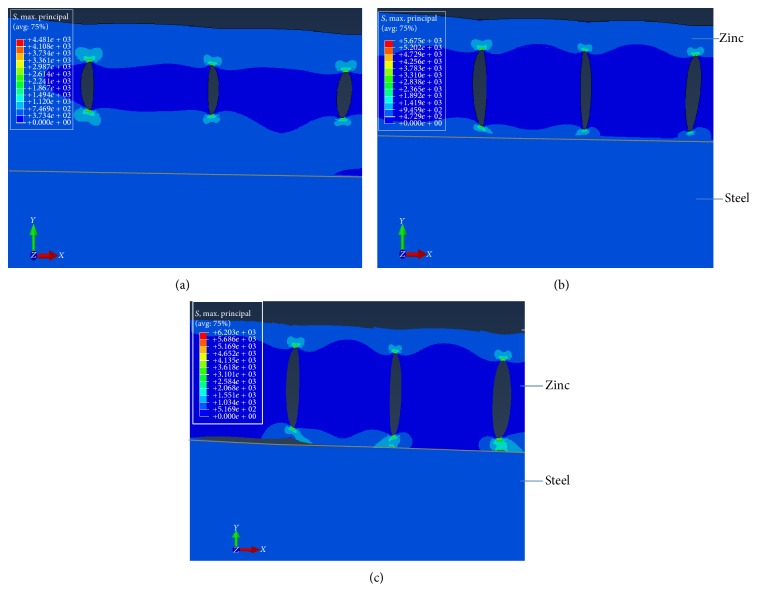
Results of FEM with three cracks after a 500 MPa loading charge: (a) length of cracks equal to 30 *μ*m, (b) the cracks after propagation, when they reach 0.57 *μ*m length, and (c) the cracks forming a deflection between the steel and zinc.

(b) Model with two or more cracks: in the second assumption, a “multiple cracks” model, namely, with 3 cracks, was implemented using almost the correspondent algorithm but specifying 3 initial cracks with an initial size of 30 *μ*m for each crack, and then each crack would increase by 3 *μ*m, up to the maximum size of 60 *μ*m. Thus, the maximum cracks value is assigned when the cracks reach the steel-zinc interface. In this model with 3 cracks the applied load (500 MPa) was considered as statement from the case of the model with a single crack. The motivation to implement a model with three cracks aims to detect whether the situation changed due to the increased (crack) stress intensity factor (SIF) values, as a result of the cumulative energy developed by these 3 cracks. Related work has been reported by Song et al. [14] who declared that the interface crack will propagate if the principal maximum stress at the crack tip exceeds a critical value. The configuration involved in this assumption is presented in Figure 13. Further, the computation results were sketched in Figure 14.

**Figure 15:**
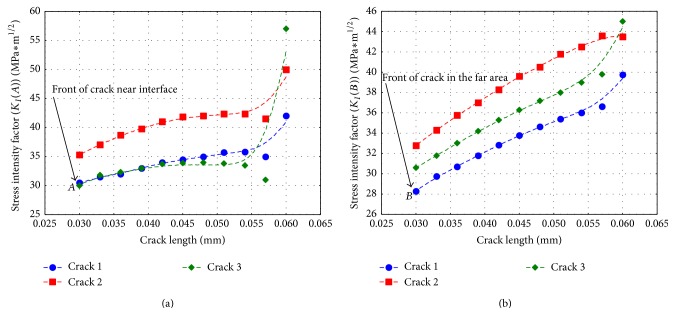
Value of the SIF for three cracks in the bimaterial: (a) value of SIF in front of crack *A* during propagation and (b) value of SIF in front of crack *B*.

Thus, confirming our initial supposition, the results presented in Figure 14 show an increase for maximum stress value within front of the cracks. At the same time, the surface denominated “debonding area” appears more pronounced. The tendency was confirmed from the development of the (crack) stress intensity factors (SIF), *K* values, where the results are reported in Figure 15. The shapes of the curves in Figure 15 show a similar trend obtained by the one in the case with one crack except for the fact that “crack 2” shows the highest influence of propagation, derived from the cumulated extension efforts from another two cracks. Another thing to note is the gap difference of SIF for crack lengths 57 *μ*m and 60 *μ*m. This difference could be explained by the appearance of the previously mentioned “debonding areas” (i.e., imposed length for debonding area considered in this research was a maximum of two initial crack lengths, because after this size the peeling processes occur). Although the difference is evident, it does not exceed the critical value of Mode I fracture toughness; for example, the value cited by Ashby [15] for steel is about 80–170 MPa m^0.5^. Consequently, the crack will not cross into the steel material and in the worst case the crack/cracks will form a large bifurcation at the interface between these two materials.

## 6. Conclusion

This research paper highlights by means of experimental, numerical, and analytical tool the behaviour of a bimaterial zinc/steel interface submitted to mechanical loading. In particular, the case of crack/cracks at the bimaterials interface was considered. The main objectives of this paper were to assess the behaviour of the crack/cracks generated at the end of the hot-dip process and to reveal that the numerical computations are in a good agreement with the experimental outcomes.

Optical microscopy and scanning electron microscopy (SEM) techniques were involved to evaluate the experimental performance of the bimaterials compound. Postmortem analysis of the fracture surfaces emphasized the uniformity of the zinc layer over the steel surface and provided accurate information related to the average of cracks length (Figure 4). In addition, this technique allows quantifying the number of cracks (Figure 3) that forms in the zinc layers. In the meantime, this assessment provides patterns about the evolution of crack from incipient phase, where the cracks arise, and how far they spread.

In numerical computations, simulations of the model considered were run in two different situations, namely, when the bimaterial contains just one crack or else 3 cracks, in order to prove the agreement between computation and experimental outcomes. Besides, to get even accurate results, the analytical model used by Erdogan et al. [7] was also confronted. The SIF were calculated to obtain value of the stress singularity that characterizes the crack evolution in the bonding area of bimaterial, for both crack tip values, that is, the fronts of cracks *A* and *B*.

The analytical, numerical, and experimental assessments prove that the crack/cracks that arise during the hot-dip galvanized steel process will propagate during the mechanical fatigue test. It was also established that the crack/cracks will stop near the steel-zinc interface at a distance of about 3 *μ*m and only in particular cases yield a deflection at the bimaterial interface.

To summarize, the results may confirm that cracks reduce the life span of the bimaterial but are not responsible for a direct degradation of the steel. This method is therefore useful for employment in damage models that include the safety factor condition. Finally, a nondestructive method should be employed to detect the behaviour of bimaterials for a better calibration.

## Figures and Tables

**Table:** (a) Mechanical properties

Material		S420MC (brut)	HE360DR (brut)	S420MC (galvanized)	HE360DR (galvanized)
Properties	Yield tensile strength (MPa)	366	460	450	514
	Ultimate tensile strength (MPa)	466	574	455	539

**Table:** (b) Chemical analysis

	C (%)	Mn (%)	P (%)	S (%)	Si (%)	Al (%)	Nb (%)	V (%)	Ti (%)
S420MC	≤0.12	≤0.16	≤0.025	≤0.015	≤0.50	≤0.015	≤0.090	≤0.20	≤0.15
HE360DR	0.11	1.40	0.030	0.025	0.50	0.015–0.080	0.100	0.100	0.100

**Table 2:** Number of cracks per unit of length.

Material	Average value of number of cracks per unit of length
He360DR B9	6 ± 3
He360DR H8	11 ± 2
S420MC B7	7 ± 3
S420MC H2	8 ± 2
